# Carotid sinus nerve stimulation attenuates alveolar bone loss and inflammation in experimental periodontitis

**DOI:** 10.1038/s41598-020-76194-z

**Published:** 2020-11-06

**Authors:** Aline Barbosa Ribeiro, Fernanda Brognara, Josiane Fernandes da Silva, Jaci Airton Castania, Patrícia Garani Fernandes, Rita C. Tostes, Helio Cesar Salgado

**Affiliations:** 1 Department of Physiology, Ribeirão Preto Medical School, University of São Paulo, Avenida dos Bandeirantes, 3900, Ribeirão Preto, SP 14049-900 Brazil; 2 Department of Pharmacology, Ribeirão Preto Medical School, University of São Paulo, Ribeirão Preto, SP Brazil; 3 University Center North Paulista (Unorp), São José do Rio Preto, SP Brazil

**Keywords:** Immunology, Physiology, Cardiology

## Abstract

Baroreceptor and chemoreceptor reflexes modulate inflammatory responses. However, whether these reflexes attenuate periodontal diseases has been poorly examined. Thus, the present study determined the effects of electrical activation of the carotid sinus nerve (CSN) in rats with periodontitis. We hypothesized that activation of the baro and chemoreflexes attenuates alveolar bone loss and the associated inflammatory processes. Electrodes were implanted around the CSN, and bilateral ligation of the first mandibular molar was performed to, respectively, stimulate the CNS and induce periodontitis. The CSN was stimulated daily for 10 min, during nine days, in unanesthetized animals. On the eighth day, a catheter was inserted into the left femoral artery and, in the next day, the arterial pressure was recorded. Effectiveness of the CNS electrical stimulation was confirmed by hypotensive responses, which was followed by the collection of a blood sample, gingival tissue, and jaw. Long-term (9 days) electrical stimulation of the CSN attenuated bone loss and the histological damage around the first molar. In addition, the CSN stimulation also reduced the gingival and plasma pro-inflammatory cytokines induced by periodontitis. Thus, CSN stimulation has a protective effect on the development of periodontal disease mitigating alveolar bone loss and inflammatory processes.

## Introduction

Periodontitis is a chronic inflammatory disease with high prevalence rates, representing a major public health problem^1^. It is associated with the progressive destruction of the supporting structures of the teeth (alveolar bone, periodontal ligament, and cementum) by overactivity of host immune-inflammatory agents in response to dysbiosis biofilm^2,3^. The host responds to microbial challenge activating components of innate and adaptative immunity, followed by the production of inflammatory mediators and generation of an overwhelming pro-inflammatory response^4^. Pro-inflammatory cytokines are produced by resident cells (epithelial cells, gingival and periodontal ligament fibroblasts, osteoblast, and dendritic cells), phagocytes (neutrophils and macrophages) and lymphocytes. Tumor necrosis factor-alpha (TNFα), one of these pro-inflammatory cytokines, induces inflammatory cells migration to tissue destruction^5^, upregulates the release of interleukin-1 beta (IL-1β) and interleukin-6 (IL-6)^6,7^, and increases osteoclastogenesis^8,9^. The osteoclastogenesis is also controlled by the autonomic nervous system^10,11^. The sympathetic nervous system acts as a negative bone mass regulator, inhibits the proliferation of osteoblasts and promotes osteoclastogenesis^12^. In contrast, the parasympathetic nervous system acts positively in the control of bone mass, promoting apoptosis of osteoclasts^11,13^.

Experimental and clinical evidence indicates an association between the autonomic nervous system and the immune response^14,15^. The brain inhibits inflammation through three pathways: the activation of the hypothalamic–pituitary–adrenal axis releasing glucocorticoids^16^; the cholinergic vagal anti-inflammatory pathway^17^; and the sympathetic splanchnic anti-inflammatory pathway^18^. Over the past twenty years, many studies were performed to better understand the relationship between the immune system and both branches (parasympathetic and sympathetic) of the autonomic system^17,19–21^. Our research group reported that baroreflex activation, through electrical stimulation of the aortic depressor nerve, attenuates the joint (femorotibial)^22^ and neural (hypothalamus) inflammation in conscious endotoxemic rats^23^. The simultaneous activation of both the carotid baroreflex and peripheral chemoreflex, by electrical stimulation of the carotid sinus nerve (CSN) and carotid sinus, was very effective controlling the innate immune response induced by lipopolysaccharide^24^. It is important to highlight that the carotid sinus electrical activation has been used to treat hypertensive patients resistant to pharmacological therapy^25,26^ and also patients with heart failure^27^. Activation of the carotid baroreflex elicits sympathetic activity inhibition and parasympathetic drive activation to the heart^28^. On the other hand, peripheral chemoreflex activation leads to concomitant sympathetic and parasympathetic activation^29^. Therefore, it is reasonable to expect that simultaneous baroreflex and chemoreflex activation produces a significant anti-inflammatory response, inhibiting innate immune system, attenuating the release of pro-inflammatory cytokines and decreasing osteoclastogenesis in other inflammatory diseases, such as periodontitis.

Our laboratory developed a technique to simultaneously stimulate the carotid baroreflex and chemoreflex in unanesthetized rats^30^. This electroceutical approach allows the investigation of autonomic modulation without the undesirable effects of anesthesia under different protocols, particularly those involving inflammatory models^24^. Although the effectiveness of this approach in controlling systemic inflammation has already been described^24^, the influence of simultaneous electrical stimulation of the chemo- and baroreflex, through the CSN stimulation, on periodontitis-associated inflammatory response has not previously been investigated. It is worth to highlight that the impact of periodontal disease (PD) is not restricted to the oral cavity. PD also affects the overall health of individuals^31^ being associated with cardiovascular diseases, such as arterial hypertension, myocardial infarction, stroke, and atherosclerosis^32–35^. The present study determined the modulatory effects of long-term (9 days) carotid baroreflex and chemoreflex activation on bone resorption, histological damage and inflammatory mediators in rats submitted to ligature-induced periodontitis.

## Material and methods

### Animals

Adult male Wistar Hannover rats weighing about 250 g were obtained from the breeding facility of the University of São Paulo at Ribeirão Preto. The animals were maintained under controlled temperature (24 °C), constant 12 hours (h) light–dark cycle, while food and water were provided ad libitum*.* The rats were divided into four groups: I: SHAM + Control (fictitious surgery for ligation around the right and left first molars associated with placement of the electrodes around the CSN, but without electrical stimulation); II: SHAM + CSN (fictitious surgery for ligation around the right and left first molars associated with placement of the electrodes plus electrical stimulation of the CSN); III: PD + Control (surgery for ligation around the right and left first molars associated with placement of the electrodes around the CSN, but without electrical stimulation); IV: PD + CSN (surgery for ligation around the right and left first molars associated with placement of the electrodes plus electrical stimulation of the CSN). All experimental procedures were performed following the “Guide for the Care and Use of Laboratory Animals” prepared by the National Academy of Sciences and published by the National Institutes of Health^36^. This study was also approved by the Ethics Committee of the Ribeirão Preto Medical School, University of São Paulo, São Paulo, Brazil (protocol number 252/2017).

### Surgical procedures

Rats were anesthetized with a mixture of Ketamine (50 mg/kg, i.p.) and Xylazine (10 mg/kg, i.p.) and then submitted to surgical procedures to isolate the left CSN for implantation of electrodes as previously described^30^. Briefly, the rats were subjected to ventral neck surgery, and the left CSN was carefully isolated. The CSN received a bipolar stainless-steel electrode positioned around the left carotid sinus and CSN. The electrode was covered with silicone impression material (Kwik-Sil silicone elastomer; World Precision Instruments, Sarasota, Florida, USA). The ends of the electrode wires were conveyed subcutaneously to the interscapular region of the rats and welded to small outlets. Then, the incision in the cervical area was sutured. Control rats underwent surgery with procedures similar to those described above, but electrodes were not placed on the CSN and carotid sinus.

In the same surgery, a 4–0 sterile silk ligature was placed around the right and left first molars to induce the PD or not (SHAM). At the end of the surgery, all animals received a polyvalent veterinary antibiotic (Pentabiótico, 0.2 mL, i.m., Fort Dodge, Campinas, SP, Brazil) and analgesic (tramadol hydrochloride: 2 mg/kg, s.c., during 3 consecutive days). After 8 days, under anesthesia [Ketamine (50 mg/kg, i.p.) and Xylazine (10 mg/kg, i.p.)], the left femoral artery was catheterized with polyethene tubing (PE-50 soldered to PE-10 polyethene tube; Intramedic, Clay Adams, Parsippany, NJ, USA) for arterial pressure recording.

### Assessment of the hemodynamic parameters and electrical stimulation of the carotid sinus nerve

Twenty-four hours after the femoral artery catheterization, the pulsatile arterial pressure was recorded in unanesthetized freely moving animals placed in individual cage. The arterial catheter was connected to a pressure transducer (MLT844; ADInstruments, Bella Vista, Australia), and the arterial pressure signal was amplified (ML224; ADInstruments, Bella Vista, Australia). The signal was sent to an IBM/ PC computer (Core 2 Duo, 2.2 GHz, 4 GB RAM) attached to an analogue-to-digital interface (PowerLab, ADInstruments, Bella Vista, Australia). The electrodes were connected to an external square pulse generator to stimulate the CNS [1.5–3 V; 1 ms; 30 Hz; for 10 minutes (min)]. Pulsatile arterial pressure recordings were processed with a computer software (LabChart 8.0, ADInstruments, Bella Vista, Australia) capable of detecting inflexion points, systolic, diastolic and mean arterial pressure (MAP); as well as heart rate (HR) beat-by-beat time series.

### Experimental procedures

**Figure 1:**
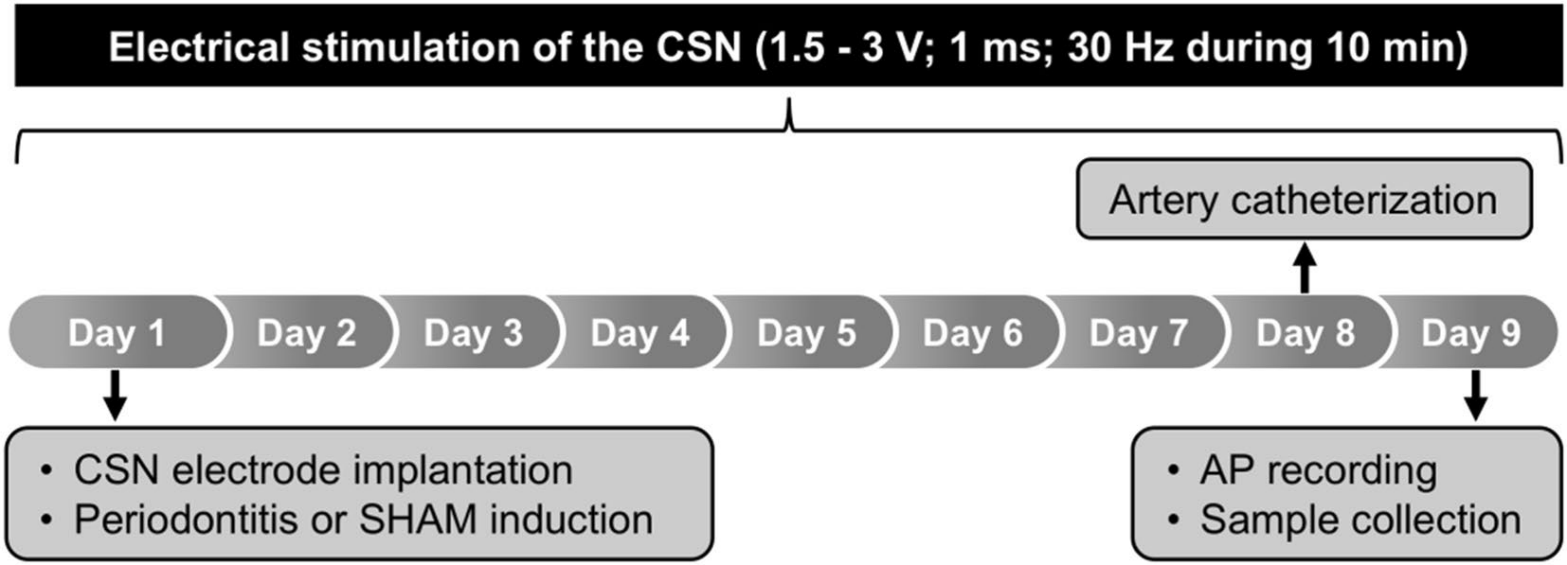
The experimental protocol. On the first day, the electrodes were implanted around the carotid sinus nerve (CSN) combined with the mandibular ligation, around the first lower molars, to induce periodontitis. Electrical stimulation of the CSN was performed for nine days, starting on the first day of dental ligation. On the eighth day, a catheter was inserted into the left femoral artery, while in the next day the arterial pressure (AP) was recorded to confirm the effectiveness of the electrical stimulation of the CSN. Next, a blood sample, the gingival tissue and the jaw were collected for further analysis. SHAM, fictitious ligation.

After the surgical procedures, electrical stimulation of the CSN was performed daily during nine consecutive days in unanesthetized rats, starting the first day of dental ligation (Fig. 1). On the ninth day, the pulsatile arterial pressure was recorded for 30 min, followed by the CSN electrical stimulation to confirm the effectiveness of the electrical stimulation. This effectiveness was confirmed by the hypotensive response caused by the CSN electrical stimulation. Then, a blood sample (1 mL), gingival tissue (right and left) and the jaw (separated on the right and left) were collected. The blood samples were maintained at 4 °C and centrifuged for 20 min at 3,500 rpm. Then, the plasma was frozen at -80 °C for further analysis of inflammatory cytokines. The gingival tissue was transferred to a microtube with RNALater reagent (Ambion, Austin, TX, USA) and stored at 4 °C during 24 h and then stored at -80 ºC.

### Micro-computed tomography analysis

Non-demineralized specimens were scanned by a cone-beam micro-CT system (Skyscan 1176; Bruker, Belgium). The X-ray generator was operated at 50 kV with a source current of 500 µA and a 0.5 mm aluminum filter. A volume of interest from the apexes of all roots of the mandibular first molar (M1) up to the roof of the furcation of M1, touching the roots surfaces (CT-Analyzer; Bruker, Belgium) was selected for bone volume measurement, as previously described^37,38^. The bone volume/tissue volume (BV/TV), porosity, trabecular number (Tb.N) and trabecular separation (Tb.Sp) were analyzed in the volume of interested area with CtAn (Bruker, Belgium). The data sets were reconstructed with CTVox (Bruker, Belgium). All micro-CT analyses were performed by one masked and calibrated examiner.

### Histological analysis

The jaws were adequately fixed in 10% buffered formaldehyde, and after decalcified in 4% ethylenediaminetetraacetic acid solution, buffered with sodium phosphate, pH 7.4. After complete decalcification, the specimens were immersed into 30% sucrose solution in PBS, until tissue saturation. Serial Sects. (10 µm thick) were cut, using a cryostat, from the buccal toward the lingual direction. These sections were stained with hematoxylin and eosin for analysis by light microscopy (DM 5500B; Leica Microsystems, Wetzlar, Germany). Two sections, from each sample, representing the central buccal–lingual portion in the furcation area of the first molar, were selected for histopathologic and histometric analysis. The histopathological scores in interdental (between the first and the second molar) region were analyzed under light microscopy, as described previously^39^. The scores were assigned as follows: **score 0**, absence or sparse inflammatory cell infiltration, preserved alveolar process and cementum; **score 1**, moderate inflammatory cell infiltration in the insert gingival with intact cementum and minor alveolar process resorption; **score 2**, accentuated cellular infiltration of the gingival and periodontal ligament, and marked degradation of the alveolar process and part of the cementum; and **score 3**, accentuated cellular infiltrate in both gingival and periodontal ligament, complete alveolar process resorption and severe cementum destruction.

For the histometric alveolar bone analysis, the images of the histologic sections were analyzed using appropriate software (ImageJ 1.50i, a version of Wayne Rasband, National Institutes of Health, USA; https://imagej.nih.gov/ij). The furcation region not filled with bone was measured by the linear distance between the area surrounded to the roof of the furcation to alveolar crest in the furcation.

### Plasma cytokine measurement

The plasma concentrations of IL-6, TNFα, IL-1β and IL-10 were determined using enzyme-linked immunosorbent assay (ELISA) kits from R&D Systems (Minneapolis, MN, USA) according to the manufacturer’s instructions. The results were expressed as pg/mL, based on standard curves.

### Analysis of gene expression in gingival tissue

Briefly, the total RNA from gingival tissue was extracted using the RNeasy Plus Mini Kit (Qiagen, Hilden, Germany), and 500 ng of total RNA was reverse-transcribed using the QuantiNova Reverse TraNSCription kit (Qiagen, Hilden, Germany) according to the manufacturer’s kit instructions. The reactions were performed using TaqMan Gene Expression Assays (Thermo Fischer Scientific, Waltham, MA, USA), according to the manufacturer’s recommendations and TaqMan Universal Master Mix II (Thermo Fischer Scientific, Waltham, MA, USA). Undetermined values in SHAM groups were set to a maximum Ct (e.g. 40), because the majority were non-detects, as previously suggested^40^. Data were analyzed by the 2^-ΔΔ*C*t^ method^41^, and the results expressed in relation to the relatively to control. The data were represented as the difference (2^-ΔΔCt^) in TNFα, IL-β, IL-6 and IL-10 genes expression, which were normalized by GAPDH.

### Statistical analysis

The statistical analysis was performed using two-way analysis of variance (ANOVA) for repeated measures, followed by the Tukey’s multiple comparisons post-hoc test. The data obtained from the plasma, gingival tissues and histopathological score were statistically analyzed using the non-parametric Kruskal–Wallis test followed by the Dunn post-test to compare medians. Values are expressed as the mean ± standard error of the mean (SEM). Differences were considered significant at *P* < 0.05.

## Results

### Hemodynamic responses to CSN electrical stimulation

**Figure 2:**
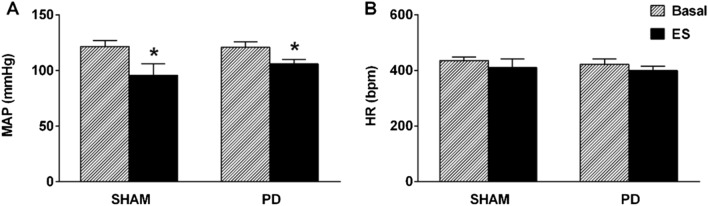
CSN stimulation elicits a hypotensive response. Mean arterial pressure (MAP; **A**) and heart rate (HR; **B**) at baseline and during electrical stimulation (ES) of the carotid sinus nerve in SHAM (n = 5) and periodontal disease (PD) rats (n = 8). Bars represent the mean ± standard error. *P < 0.05 versus basal. CSN, carotid sinus nerve; SHAM, fictitious ligation.

The CSN electrical stimulation reduced MAP in both SHAM and PD rats (Fig. 2A). Moreover, this hypotensive response was similar between the groups (Fig. 2A). Electrical stimulation of the CSN did not change HR in SHAM or PD rats (Fig. 2B).

### Micro-computed tomography analysis of alveolar bone

**Figure 3:**
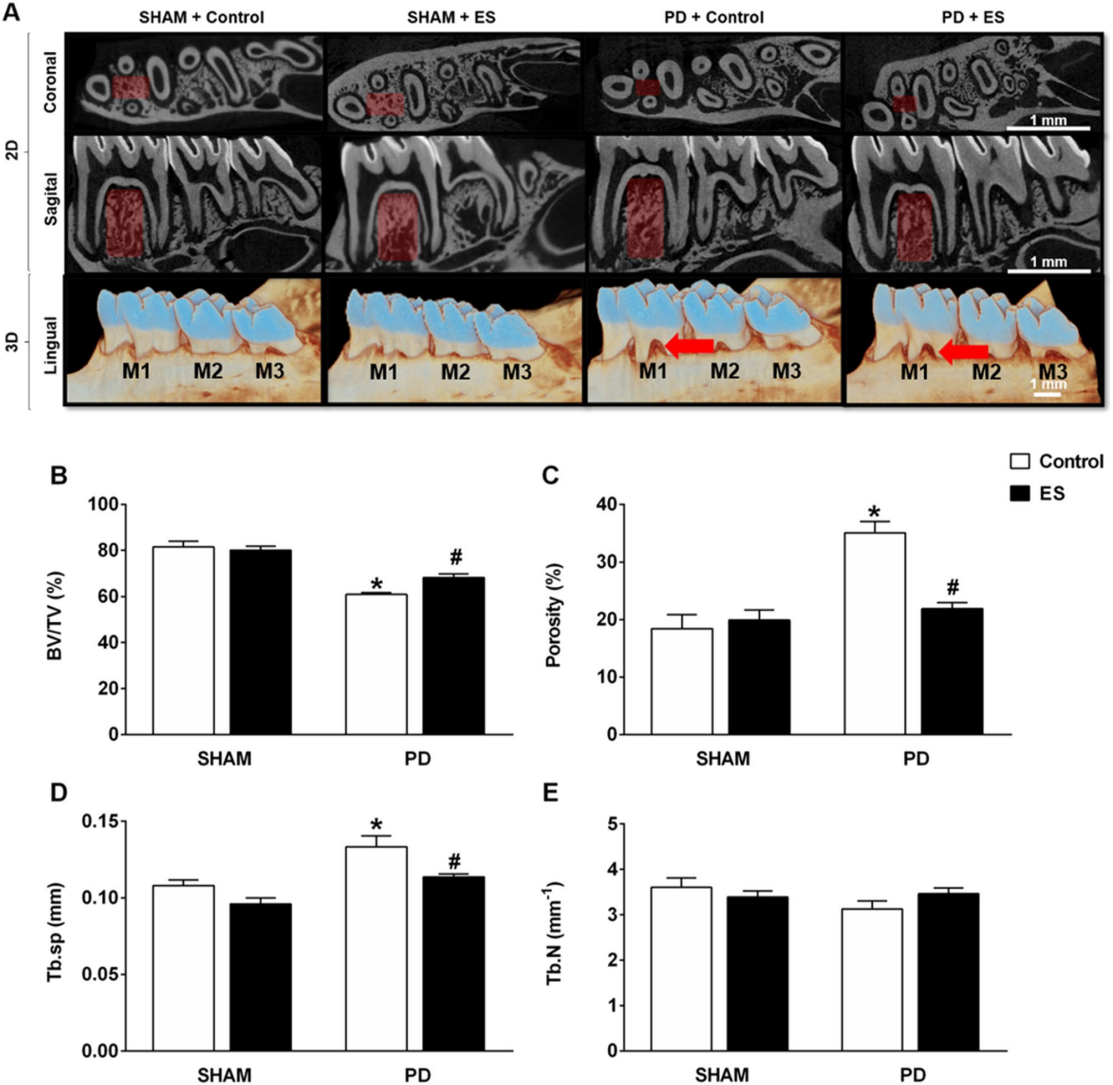
Stimulation of the CSN attenuates the bone loss induced by periodontitis, Evaluation trough 2D and 3D image analysis. Micro-CT images of the jaw (**A**) from SHAM rats with (n = 5) and without (n = 5) CSN stimulation and jaw from PD rats with (n = 8) and without (n = 6) CSN stimulation. Bar graphs represent bone volume/tissue volume (**B**; BV/TV), total bone porosity (**C**), separation of the trabeculae (**D**; Tb.Sp) and the trabecular number (**E**; Tb.N) in all evaluated groups. In Panel A, the red color arrow in the image from PD control group highlights the accentuated bone loss in the first molar (M1); the red color arrow in the image from PD plus CSN stimulation highlights the attenuation of the bone loss due to the CSN activation. In contrast, the area highlighted in dark red color indicates the bone volume of interest, where the bone volumetric analysis was performed. Bars represent the mean ± standard error. *P < 0.05 versus SHAM + Control; #*P* < 0.05 versus PD + Control. ES, electrical stimulation; M2, lower second molar; M3, lower third molar; SHAM, fictitious ligation.

The bi-dimensional (coronal and sagittal) and three-dimensional (lingual) views from the micro-computed tomography of the jaw revealed a typical alveolar bone architecture, characterized by the absence of alveolar bone resorption in groups SHAM control and SHAM plus CSN stimulation (Fig. 3A). Likewise, the same analysis showed a higher bone loss in the PD control group, classically observed in PD (Fig. 3A). However, in rats with PD plus CSN stimulation, decreased alveolar bone resorption was observed compared to the PD control group (Fig. 3A).

Quantification of bone in the furcation area showed no changes in the SHAM groups (stimulated or not) (Fig. 3B–E). However, the PD control rats exhibited a decrease in bone volume/tissue volume (BV/TV; Fig. 3B), an increase in porosity (Fig. 3C), and trabecular separation (Tb.Sp; Fig. 3D), compared to all the other groups. On the other hand, CSN stimulation attenuated the bone loss induced by PD (Fig. 3B–D). There was no difference in Th.N (Fig. 3E) among the groups.

### Histological analysis of the effects of CSN stimulation

**Figure 4:**
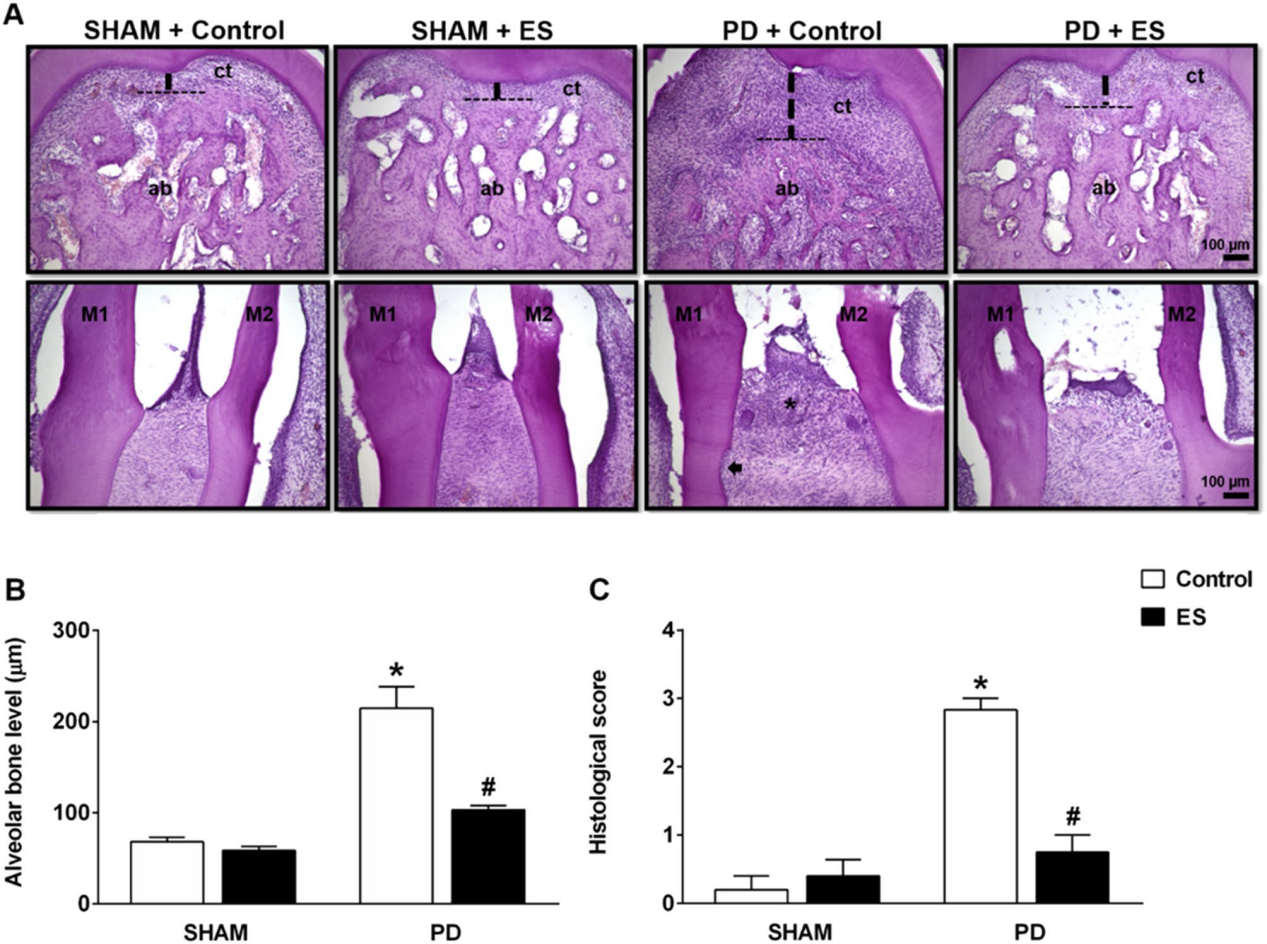
Stimulation of the CSN reduces bone loss and histological damage induced by periodontitis. Histological images of the jaw (**A**) from SHAM rats with (n = 5) and without (n = 5) CSN stimulation and from PD rats with (n = 8) and without (n = 6) CSN stimulation. The horizontal interrupted lines in the furcation area indicate the linear measurements of bone loss; the asterisk (*) indicates infiltration of inflammatory cells, and arrow indicates the loss of cementum. Bar graphs represent linear alveolar bone loss (**B**), and histological score (**C**) represented by bone loss, infiltration of inflammatory cells, and loss of cementum. Quantifications were performed using ImageJ 1.50i software, a version of Wayne Rasband, National Institutes of Health, USA (https://imagej.nih.gov/ij). Bars represent the mean ± standard error. *P < 0.05 versus SHAM + Control; #*P* < 0.05 versus PD + Control. CSN, carotid sinus nerve; ES, electrical stimulation; PD, periodontal disease; SHAM, fictitious ligation; ct, connective tissue; ab, alveolar bone; M1, lower first molar; and M2, second lower molar.

The histological analysis of the excised jaw showed no differences in the alveolar bone level or histological score between the SHAM groups (Fig. 4). Nevertheless, PD control rats exhibited severe inflammatory cellular infiltration, process resorption and severe cementum destruction (Fig. 4A), which was reflected by increased alveolar bone loss levels (Fig. 4B) and histological scores (Fig. 4C) compared to other animals. However, the periodontium of the rats with PD plus CSN stimulation showed considerable preserved alveolar process, well-preserved cementum, and reduced cellular influx (Fig. 4A–B), resulting in lower histological scores (Fig. 4C).

### Effects of CSN stimulation on plasma cytokines concentration

**Figure 5:**
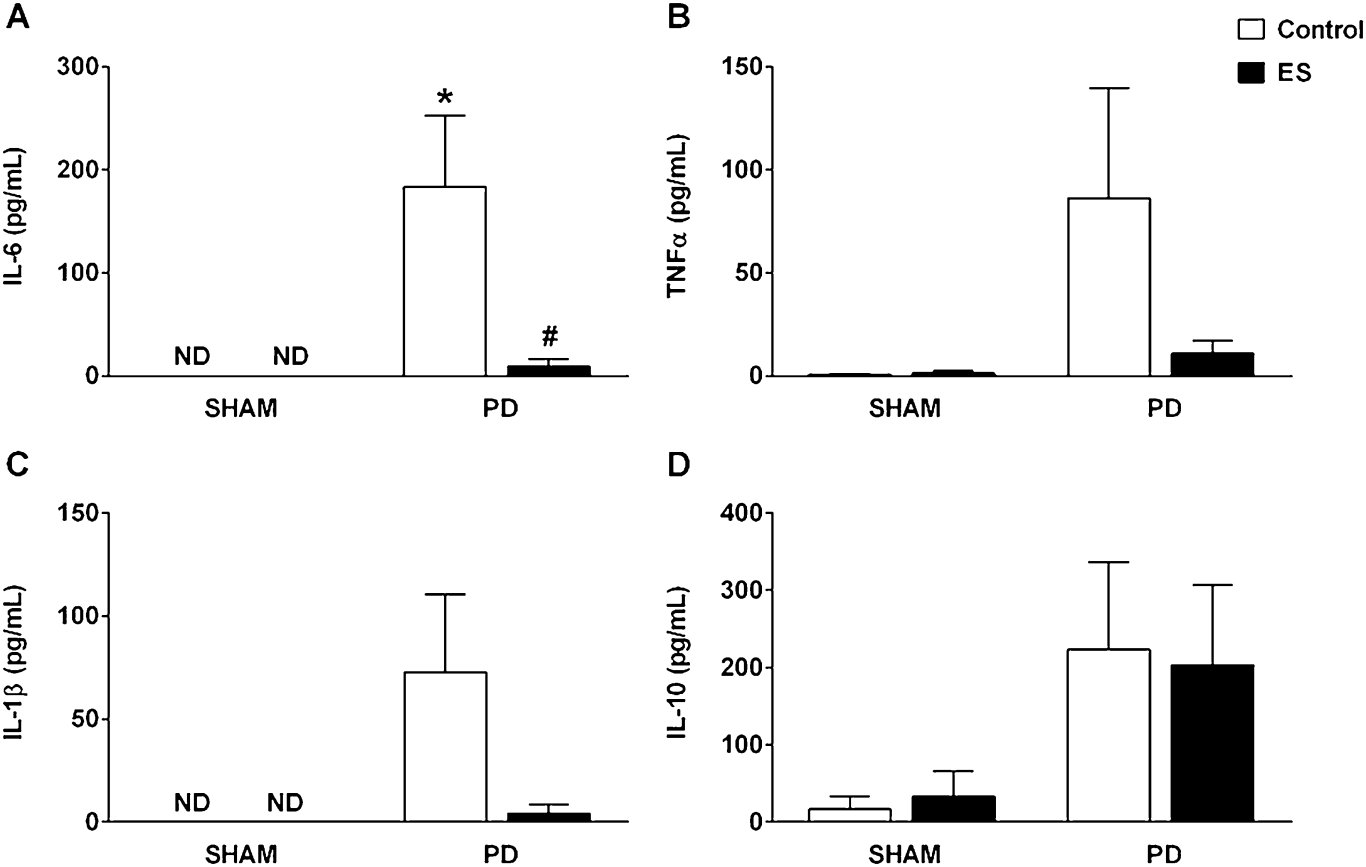
CSN activation decreases plasma levels of IL-6. Plasma concentrations of IL-6 (**A**), TNFα (**B**), IL-1β (**C**) and IL-10 (**D**) of SHAM rats with (n = 5) and without (n = 5) CSN stimulation and of PD rats with (n = 8) and without (n = 6) CSN stimulation. Bars graphs represent the mean ± standard error. *P < 0.05 versus SHAM + Control; #*P* < 0.05 versus DP + Control. CSN, carotid sinus nerve; ES, electrical stimulation; ND, not detected; PD, periodontal disease; SHAM, fictitious ligation.

In the PD control group, high levels of the proinflammatory cytokine IL-6 were detected in plasma (Fig. 5A). Nevertheless, CSN stimulation reduced the levels of IL-6 induced by PD in the plasma (Fig. 5A). Plasma levels of IL-6 and IL-1β were not detected in SHAM plus CSN stimulated or SHAM control groups (Fig. 5A,C). Moreover, no differences were observed in plasma levels of TNFα, IL-1β or IL-10 among groups (Fig. 5B–D).

### Effects of CSN stimulation on gingival cytokines expression

**Figure 6:**
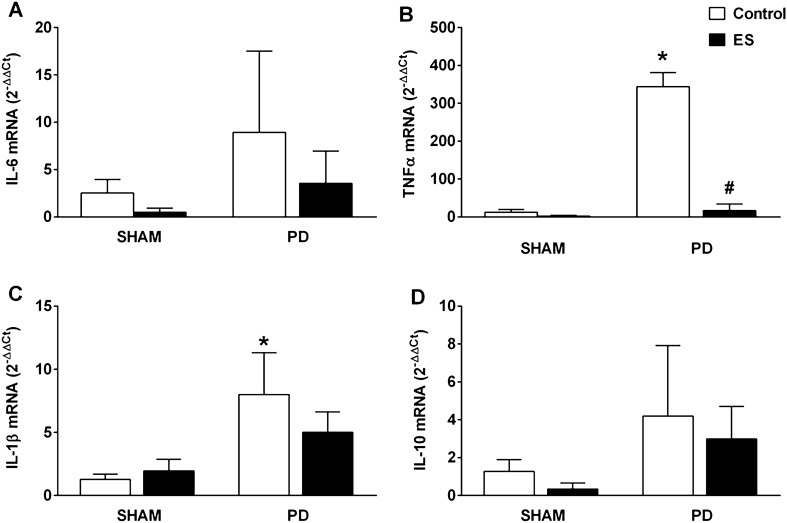
CSN stimulation decreases the expression of TNF*α* mRNA expression in gingival tissues. Expression of IL-6 (**A**), TNFα (**B**), IL-1β (**C**) and IL-10 (**D**) mRNA in gingival tissue of SHAM rats with (n = 3) and without (n = 3) CSN stimulation and of PD rats with (n = 4) and without (n = 3) CSN stimulation. Gene expression values are in relation to the SHAM group without stimulation of the CSN (2^−ΔΔCt^), which was normalized by GAPDH. Bars graphs represent the mean ± standard error. *P < 0.05 versus SHAM + Control; #*P* < 0.05, versus DP + Control. CSN, carotid sinus nerve; ES, electrical stimulation; PD, periodontal disease; SHAM, fictitious ligation.

No changes were found in the expression of IL-6 and IL-10 in the gingival tissue among the groups (Fig. 6A,D). However, in addition to alveolar bone loss and histological damage, the ligature induced in the control rats a higher expression of TNFα and IL-1β in the gingival tissue, compared to the SHAM groups (Fig. 6B,C). Importantly, mRNA expressions of gingival TNFα decreased in the PD rats submitted to CSN stimulation when compared to the PD plus control rats (Fig. 6B). Nevertheless, the same was not observed with respect the IL-1β levels (Fig. 6C).

## Discussion

The present study demonstrates, for the first time, that long-term (9 days) CSN electrical stimulation (nine days) in unanesthetized rats attenuates bone loss, histological damage between the first and the second molar, local (gingival) and systemic (plasma) pro-inflammatory cytokines induced by periodontitis. Thus, these results indicate that sympathetic and parasympathetic activation, through the CSN stimulation, can prevent the damage of the ligature-induced periodontitis in rats.

The autonomic nervous system influences the periodontal tissue^42^. Autonomic nerve fibers were found in the cortical bone, endosteum, and periosteum, suggesting a direct effect on bone cell function^43,44^. Moreover, the sympathetic activity inhibits the proliferation of osteoblasts and promotes bone loss trough the norepinephrine release^45,46^. In contrast, parasympathetic activity has anti-inflammatory properties and decreases bone resorption by suppressing the sympathetic activity and increasing the apoptosis of osteoclasts^13,47^. Acetylcholine, the principal neurotransmitter released by the parasympathetic nervous system^48^, is synthesized by periodontium cells, as well as cells of the immune system, such as monocytes/macrophages, B and T cells^49–51^. Previous studies showed that acetylcholine has anti-inflammatory activity through the activation of the alpha 7 nicotinic receptor, acting on immune-competent cells regulating inflammatory processes^20,21^. The cholinergic anti-inflammatory pathway has been extensively investigated with therapeutic purposes to treat chronic inflammatory diseases, such as rheumatoid arthritis, asthma, sepsis, diabetes and psoriasis^52^. However, the effect of this pathway in the treatment of PD is currently unknown. It is possible that the beneficial effects observed in bone loss and inflammation in PD, caused by the simultaneous stimulation of the carotid baroreflex and chemoreflex described in the present study, maybe due to parasympathetic activation. This mechanism may protect the alveolar bone loss by decreasing the inflammation, the osteoclastogenesis and the sympathetic activity induced by PD.

Placement of a ligature around the posterior teeth mimics the development of the human periodontal disease, leading to local cellular inflammatory accumulation, apical migration of junctional epithelium, and bone loss^9,53^. The development of periodontitis is associated with dysbiotic plaque biofilms and inflammatory response mediated by the host^54^. The inflammatory response results in progressive destruction of the supporting structures of the teeth and loss of periodontal attachment^4^. Therefore, the primary outcome of periodontitis includes alveolar bone loss^4^. Beyond alveolar bone loss and gingival inflammation, the literature documents that the production of cytokines in PD can act systemically through oral bacteria and immune cells translocation in the bloodstream reaching distant organs; eliciting, therefore, a generalized inflammatory response^55^. Nevertheless, systemic inflammatory disease impacts the periodontal tissues, such as diabetes and hypertension^56,57^. This notion might explain why the physiopathogenesis of PD is similar, in nature, to other inflammatory diseases. Thus, the ligature experimental periodontal disease model has been widely used in the literature to investigate the pathophysiology and new potential therapeutic strategies for periodontitis^9^.

In the current study, rats with PD submitted to CSN activation showed lower plasma concentrations of IL-6 compared to the PD control group. IL-6 is a pro-inflammatory cytokine usually increased in periodontitis patients, and it has been likewise associated with the augmented incidence of myocardial infarction and mortality^58^. Also, PD is associated with cardiovascular diseases^32–35^, and inflammation seems to be the most likely outcome that correlates PD with cardiovascular diseases^59^. Thus, there is a connection between these three conditions: inflammation, cardiovascular diseases and PD. It has been shown that electrical stimulation of the baroreflex is an effective therapeutic approach for cardiovascular diseases, for instance, arterial hypertension and heart failure^25–27^; nevertheless, this electroceutical approach has also attenuated the inflammation in experimental PD, as demonstrated in the present study. Thus, the activation of the carotid baroreflex and chemoreflex in those clinical situations (hypertension and heart failure) would be helpful to counteract the undesirable effects of periodontitis themselves, eventually associated with those morbidities. For this reason, the results of the current study must be cautiously interpreted and carefully extrapolated to a clinical context.

In addition, in the current study, the PD control group exhibited an increase in inflammatory cell infiltration between the first and the second molar and augmentation of pro-inflammatory mediators (IL-1β and TNFα) along with the alveolar bone loss (volume and linear level). However, the levels of anti-inflammatory cytokine IL-10 and pro-inflammatory IL-6 were closer to the normal range. The increased levels of inflammatory cell infiltration and pro-inflammatory cytokines, and the alveolar bone loss are, in fact, a hallmark of periodontitis^2–4,60^. Nevertheless, the CSN stimulation in the PD reduced the alveolar bone loss and histological damage, combined with a decrease in inflammatory cells in the interproximal area between the first molar and the second molar; nevertheless, with preservation of the cementum covering the dentin.

The TNFα is one of the essential pro-inflammatory cytokines involved in the development of PD. It elicits the migration of inflammatory cells, increasing the production of IL-1β^6,7^. The increase of gingival IL-1β levels is in line with the study of Aral and co-workers (2015), that showed higher IL-1β in the ligature model of PD^60^. The over-expression of TNFα and IL-1β increases the damage of the periodontal tissue and alveolar bone loss by stimulating osteoclastogenesis and inhibiting the osteoblasts function^6,7^. The CSN electrical stimulation decreased the TNFα expression in the gingival tissue of rats, as well. It is possible that the lower expression of TNFα contributed to the reduction of bone loss in PD rats under CSN activation. In fact, previous studies showed that TNFα antagonist inhibits osteoclast formation, inflammatory response, and bone loss in experimental periodontitis^61–63^.

Baroreceptors are mechanoreceptors that monitor the arterial pressure and heart rate from the aortic arch, carotid sinuses and major blood vessels^28^. In the periodontal ligament, there is also mechanoreceptors which respond to strength application^64^. Besides, it has been suggested that the periodontal sensory innervation may interact with immunocompetent cells to assist their migration to inflamed areas of the periodontal ligament^65^. Periodontal mechanoreception is similar to baroreceptor function, and can be considered an important reflex mechanism^66^. The therapeutic method of electric activation of the baroreflex is described in the literature as Baroreflex Activation Therapy and has been used to treat some cardiovascular diseases, i.e. resistant hypertension and heart failure^25–27^. Our laboratory developed a technique to electrically stimulate the CSN in unanesthetized rats, providing simultaneous carotid baroreflex and chemoreflex activation without the undesirable effects of anesthesia^30^. In addition, both chemoreflex and baroreflex activation, through the CSN electrical stimulation, can also be used as an electroceutical approach to control the inflammation^24^. Despite that, little is known about the possible influence of the baroreflex and chemoreflex mechanisms, particularly their activation, on the hemodynamic parameters (MAP and HR) in PD. The findings in the present study indicated that CSN electrical stimulation promotes similar hypotensive responses in SHAM and PD rats. However, no changes were observed in HR due to the CSN stimulation in the SHAM or PD groups. Moreover, there was no difference between baseline arterial pressure or HR between rats with and without periodontitis. Therefore, these data are in line with previous studies that demonstrated that ligature-induced periodontitis did not affect arterial pressure in mice^67^, and also that the CSN electrical stimulation did not change HR in unanesthetized rats^30^.

Nevertheless, it is important to highlight that further studies are needed to elucidate: (1st) the mechanism involved in the baroreflex activation attenuating the PD development; (2nd) whether the chemoreflex stimulation decreased PD progress by the sympathetic or parasympathetic activation; (3rd) whether both baroreflex and chemoreflex are needed to control the PD progression; and (4th) whether the CSN stimulation would attenuate the PD development after its installation.

The current study had the limitation that concerns the use of antibiotics after periodontitis induction, that was administered to protect the animal of undesirable infection caused by the surgical procedure for implantation of the electrodes. Periodontitis was induced concomitantly with implant surgery to avoid another surgical stress. Moreover, the most important aspect of this particular protocol is that the development of periodontitis after the implantation of the electrodes and performance of the ligature combined with the antibiotic—without the electrical stimulation (control protocol)—was remarkable. In this sense, it was observed consistent results of alveolar bone loss, an increase of cytokines expression in the gingival tissue and plasma levels, and histopathological scores in the interdental (between the first and the second molar) region. Apropos, these results were similar to previous studies^60,68,69^ indicating that the protocol used in the current manuscript is straightforward, demonstrating the efficacy of the electrical stimulation of the carotid sinus nerve upon the physiopathological responses of periodontitis.

In conclusion, the present study demonstrated that the electrical stimulation of the CSN promotes a protective effect on PD development in unanesthetized rats. Moreover, the current findings provide new insights to understanding the link between baroreflex and chemoreflex activation on the modulation of periodontal bone loss.
